# Correlation of COX-2 and Ep-CAM overexpression in human invasive breast cancer and its impact on survival

**DOI:** 10.1038/sj.bjc.6600741

**Published:** 2003-02-18

**Authors:** G Spizzo, G Gastl, D Wolf, E Gunsilius, M Steurer, D Fong, A Amberger, R Margreiter, P Obrist

**Affiliations:** 1 Division of Haematology & Oncology, University of Innsbruck, A-6020 Innsbruck, Austria; 2 Department of Pathology, University of Innsbruck, A-6020 Innsbruck, Austria; 3 Tyrolean Cancer Research Institute, University of Innsbruck, A-6020 Innsbruck, Austria

**Keywords:** breast cancer, prognosis, COX-2, Ep-CAM

## Abstract

Recent studies have demonstrated cyclooxygenase 2 (COX-2) overexpression in various human malignancies, especially in breast cancer, where COX-2 turned out to be a predictor of poor survival. To evaluate the relation of COX-2 and Ep-CAM overexpression and its prognostic significance, we performed a retrospective study on 212 breast cancer patients with a median follow-up time of 10.5 years. Overexpression of COX-2 in tumour tissue samples was assessed by immunohistochemistry. COX-2 overexpression was found in 48.6% of the tumour samples and was predictive for poor disease-free and overall survival. Univariate analysis revealed a strong correlation between COX-2 and Ep-CAM overexpression (*P*=0.009). Concurrent COX-2 and Ep-CAM overexpression was present in 21.7% of tumour specimens and had an additive negative impact on disease-free and overall survival. Determination of both tumour markers should help in guiding new therapeutic strategies in patients with invasive breast cancer.

Cyclooxygenase-2 (COX-2) is a prostaglandin synthase that catalyses the synthesis of prostaglandin G_2_ (PGG_2_) and PGH_2_ from arachidonic acid. Recent studies have led to the recognition of the importance of COX-2 in tumorigenesis of different tumour types. It has been shown that COX-2 is involved in tumour angiogenesis (Tsujii *et al*, 1998; Gately, 2000), in suppression of apoptosis (Sheng *et al*, 1998) and in the promotion of invasiveness (Tsujii *et al*, 1997). COX-2 overexpression was found in pancreatic (Molina *et al*, 1999; Okami *et al*, 1999; Kokawa *et al*, 2001), oesophageal (Zimmermann *et al*, 1999), prostate (Yoshimura *et al*, 2000), lung (Khuri *et al*, 2001), head and neck cancers (Chan *et al*, 1999) and in malignant gliomas (Shono *et al*, 2001). Tsujii *et al* reported that COX-2 overexpression in intestinal epithelial cells leads to downregulation of adhesion molecules (i.e. cadherins), resulting in an enhanced tumorigenic potential (Tsujii and DuBois, 1995).

Enhanced COX-2 expression in breast cancer was first indicated by reports of elevated prostaglandin levels in breast carcinomas (Bennett *et al*, 1977), particularly in patients with metastatic disease (Rolland *et al*, 1980). A key role of COX-2 for the initiation and progression of breast cancer is suggested by the finding that mere overexpression of COX-2 can be sufficient for inducing mammary gland tumorigenesis in transgenic mice (Liu *et al*, 2001). Notably, in human breast cancer cell lines, a positive correlation was found between invasiveness, metastatic potential and prostaglandin production (Liu and Rose, 1996). Different groups have described the prognostic significance of COX-2 overexpression in breast cancer (Hwang *et al*, 1998; Ristimaki *et al*, 2002; Soslow *et al*, 2000).

We have recently described the prognostic significance of Ep-CAM overexpression in patients with invasive breast cancer (Gastl *et al*, 2000). Ep-CAM (also called 17-1A, ESA, EGP40, 323/A3) is a 40-kDa transmembrane glycoprotein expressed on most human epithelial cells (Gottlinger *et al*, 1986). The Ep-CAM glycoprotein functions as a homotypic intercellular adhesion molecule (Litvinov *et al*, 1994) and has become a target for antibody-mediated immunotherapy with the murine monoclonal antibody edrecolomab (Riethmuller *et al*, 1998). So far, no data have been reported on the correlation of COX-2 overexpression with Ep-CAM overexpression in human breast cancer. We therefore examined COX-2 and Ep-CAM overexpression in tumour specimens from 212 patients with invasive breast cancer, and analysed the prognostic value of both tumour markers.

## PATIENTS AND METHODS

### Patient selection

A total number of 212 patients were included in this retrospective analysis. This patient sample represents one-third of all cases with localised invasive breast cancer who were operated at the Department of Surgery, Innsbruck University Hospital, from 1980 to 1992. In fact, all cases for which paraffin-embedded tissue samples were still retrievable from the local pathology repository and for which clinical follow-up data were available, were included. Only patients without evidence of distant metastasis at the time of primary surgery and with well-documented axillary lymph node status were eligible for this analysis. The median age of the patients was 54.2 years (range, 29–85 years). Patients younger than 50 years were considered premenopausal. Of the women, 112 (52.8%) were node-positive and 100 (47.2%) node-negative. After primary surgery the clinical status was documented by re-evaluating each patient at least once annually at the Department of Surgery. The evaluation procedure included physical examination, mammography, abdominal ultrasound and chest radiography. The median follow-up time was 10.5 years (range, 36–240 months). During this observation period 96 patients relapsed. Of a total of 94 deaths, 84 were due to breast cancer, while 10 patients died without documented disease recurrence.

### Histopathology

All tumour samples were formalin-fixed, embedded in paraffin wax and stored at the local pathology repository. Haematoxylin- and eosin-stained slides were prepared from each tumour specimen using routine methods and then examined by light microscopy. Histologic type and tumour grade were assessed by one co-author (PO) in a blinded fashion using standard pathology criteria.

### Immunohistochemistry

COX-2 overexpression was determined by immunohistochemistry using the murine monoclonal antibody COX-2 (Cayman, USA). Briefly, 5-*μ*m sections were cut from paraffin-embedded tissue blocks, mounted on adhesive-coated glass slides, deparaffinised and rehydrated. Endogenous peroxidase was inactivated by immersing the slides in 0.3% H_2_O_2_ in absolute methanol for 20 min at room temperature. Pretreatment consisted of a 15-min incubation period in a water bath at 90°C. After washing in Tris buffer, slides were incubated for 2 h with the primary antibody (COX-2, Cayman, USA, dilution 1 : 100). Afterwards, a peroxidase-conjugated goat anti-mouse antibody ready-to-use (EnVision™, DAKO, Vienna, Austria) was added for 30 min. For immunostaining, slides were then placed into the chromogen consisting of a diaminobenzidine solution. Finally, slides were counterstained with Mayer's Hemalum solution. In addition, slides were immunostained for Ep-CAM essentially as described previously (Gastl *et al*, 2000; Spizzo *et al*, 2002).

### Evaluation of slides

COX-2 overexpression was evaluated by two independent assessors (GS and PO) using light microscopy. Reading of tissue slides was blinded, and both assessors were unaware of clinical outcome. COX-2 expression was defined as the presence of specific staining in the cytoplasm of tumour cells. A final expression score was calculated for each tissue sample by multiplying a staining intensity score (0, negative; 1, weak; 2, moderate; 3, strong staining) with a proportion score of positively stained cells (1, 1–10%; 2, 11–50%; 3, 50–80%; 4, 80–100 %). Only samples *with a final expression score* >4 were defined as ‘overexpressing’. Ep-CAM overexpression was evaluated as previously reported (Gastl *et al*, 2000; Spizzo *et al*, 2002).

### Statistical methods

Statistical analysis was performed with the SPSS software program for Windows™. The primary end points in this study were disease-free and overall survival. Thus, survival curves were calculated according to the method of Kaplan and Meier. *P*-values were evaluated using the log-rank test for censored survival data. Follow-up time was censored if the patient was lost to follow-up. Patients who died without documented disease recurrence were considered censored for disease-free survival but were included as deaths for overall survival analysis. The relation between antigen overexpression and other clinical or tumour parameters was calculated with the *χ*^2^ test. To determine the relative importance of COX-2 and Ep-CAM overexpression and established prognostic markers, these variables were subjected to multivariate analysis (Cox regression).

## RESULTS

**Figure 1:**
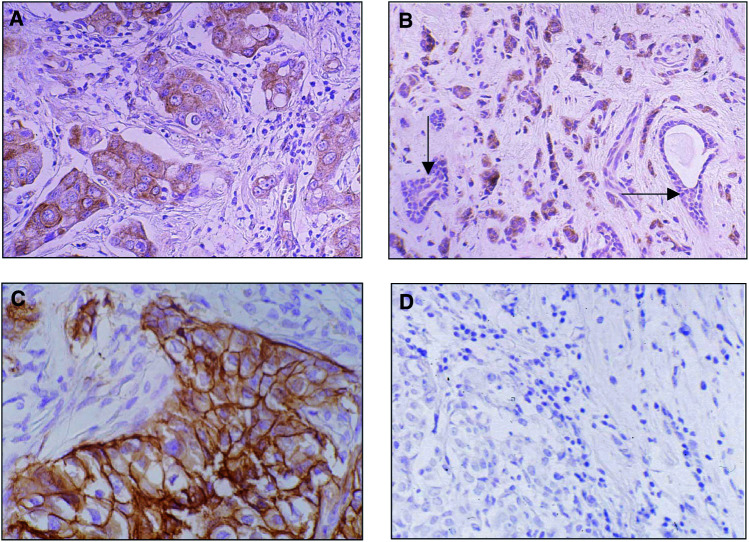
(**A**) Example of an invasive ductal carcinoma with strong cytoplasmic COX-2 staining, classified as tumour with COX-2 overexpression. (**B**) Tumour sample showing COX-2 overexpression in invasive lobular carcinoma surrounding normal epithelium lacking COX-2 expression (arrows) as internal negative control. (**C**) Example of invasive ductal carcinoma presenting with strong membraneous Ep-CAM staining, classified as tumour with Ep-CAM overexpression. (**D**) Invasive ductal carcinoma without Ep-CAM expression as negative control.

**Figure 2:**
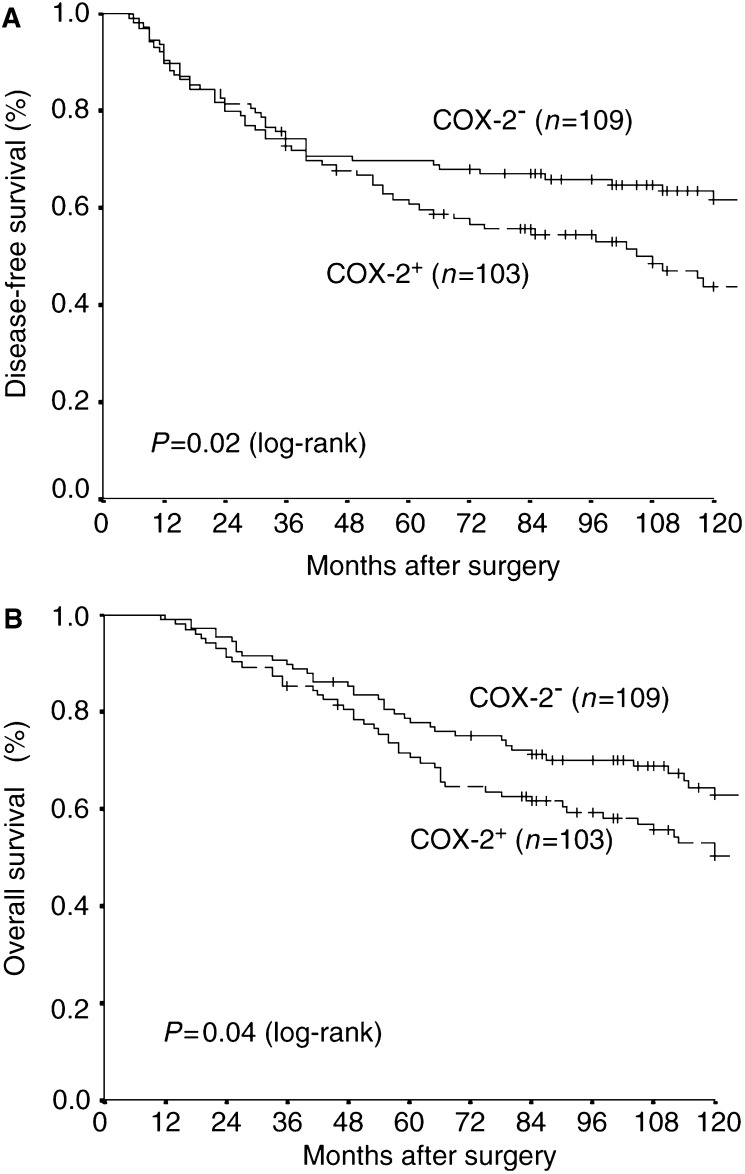
COX-2 overexpression as prognostic marker in a patient sample of 212 breast cancer patients. Patients with tumour tissue presenting COX-2 overexpression (COX-2^+^) had a significant shortened disease-free intervall (A) and overall survival (B) as compared to patients with tumours lacking COX-2 overexpression (COX-2^−^).

In normal mammary epithelium COX-2 showed absent to weak staining (Figure 1B

). COX-2 overexpression in tumour tissue (Figure 1A) was found in 103 of 212 (48.6%) tumour specimens and correlated with poor disease-free (*P*=0.02, Figure 2A

) and overall survival (*P*=0.04, Figure 2B). Remarkably, COX-2 overexpression was significantly correlated with Ep-CAM overexpression (*P*<0.009; *χ*^2^ test), histologic tumour type (*P*=0.011) and menopausal status (*p*=0.047) but failed to correlate with Her-2/neu status or other tumour parameters (Table 1

**Table 1:** Relationship of COX-2 overexpression and conventional clinical and tumour parameters

		**No**		**Yes**		
**Characteristics**	**Patients no.**	**no.**	**%**	**no.**	**%**	***P*-value^a^**
Age at diagnosis						
<50	93	55	59	38	41	0.047
⩾50	119	54	45	65	55	
						
Histological type						
Ductal	148	71	48	77	52	0.011
Lobular	45	22	49	23	51	
Other types	19	16	84	3	16	
						
Histologic grade						
I	11	8	73	3	27	0.291
II	129	62	48	67	52	
III	66	33	50	33	50	
NE	6					
						
Nodal status						
pN0	100	51	51	49	49	0.909
pN1/2/3	112	58	52	54	48	
						
Tumour size (cm)						
<2	83	44	53	39	47	0.214
2–5	99	50	51	49	49	
>5	9	2	22	7	78	
Unknown^b^	21					
						
ER						
Neg: 0–9 fmol	45	20	44	25	56	0.367
Pos: >9 mol	136	71	52	65	48	
Unknown^b^	31					
						
PR						
Neg: 0–9 fmol	62	26	42	36	58	0.105
Pos: >9 fmol	119	65	55	54	45	
Unknown^b^	31					
						
Her-2/neu						
Pos	52	22	42	30	58	0.130
Neg	160	87	54	73	46	
						
Ep-CAM						
Pos	76	30	39	46	61	0.009
Neg	136	79	58	57	42	

a χ^2^ test.

b Unknown cases are excluded from *P*-value calculation. NE=not evaluable.

**Figure 3:**
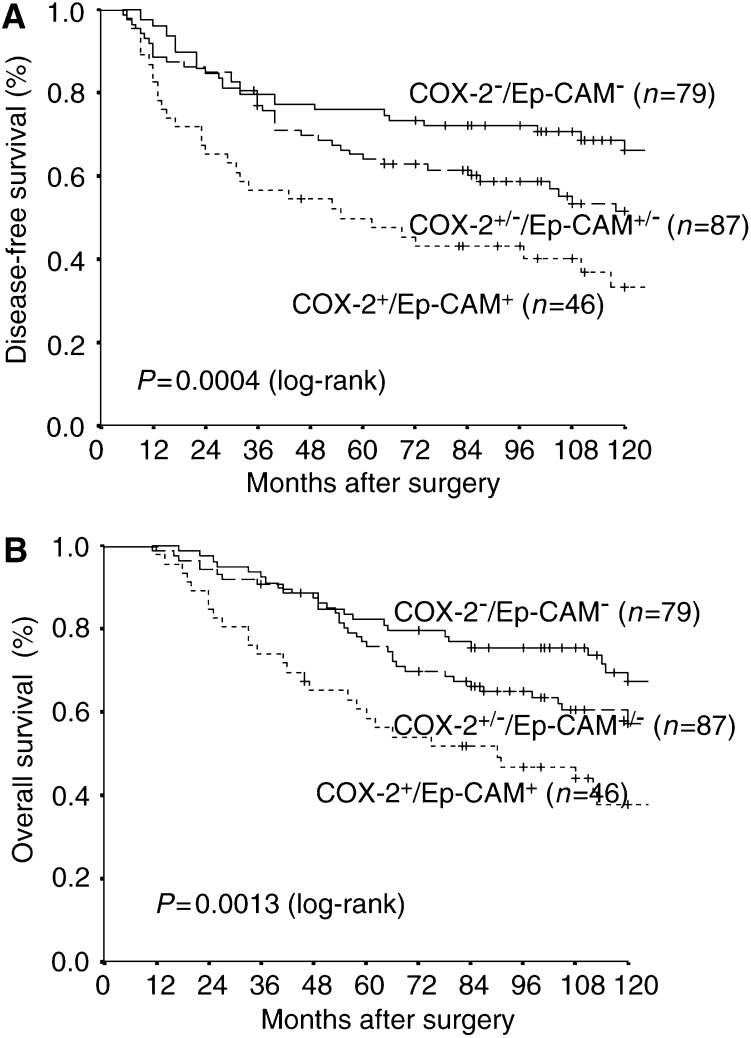
Relationship between COX-2 and Ep-CAM overexpression with disease-free survival (**A**) and overall survival (**B**). COX-2^+^/Ep-CAM^+^: patients with tumours overexpressing both antigens, with a median disease-free and overall survival of 55 and 90 months, respectively. COX-2^+/−^/Ep-CAM^+/−^: patients with tumours overexpressing only one of the two antigens, with a median disease-free and overall survival of 127 and 147 months, respectively. COX-2^−^/Ep-CAM**^−^**: patients with tumours without overexpression of the antigens, where median disease-free and overall survival were not reached.

COX-2 overexpression was significantly correlated with Ep-CAM overexpression, histologic tumour type and menopausal status, but failed to correlate with other tumour parameters.

). In 46 (21.7%) of the tumour specimens overexpression of both COX-2 and Ep-CAM was found, while 79 (37.3%) showed neither COX-2 nor Ep-CAM overexpression. Further, three distinct subgroups were identified by the expression of COX-2 and Ep-CAM antigens (Figure 3A, B

). Patients with tumours overexpressing both antigens carried the poorest prognosis. Median disease-free and median overall survival time in this patient population were 55 months and 90 months, respectively. Patients overexpressing either COX-2 or Ep-CAM had an intermediate prognosis with a median time to relapse of 127 months and a median survival time of 147 months. Finally, patients without overexpression of COX-2 and Ep-CAM in their tumours carried the best prognosis. Median time to relapse and median survival time for this patient group were not reached. By subgroup analysis, overexpression of COX-2 in node-positive cases predicted a dismal prognosis regarding disease-free and overall survival, whereas in node-negative cases COX-2 overexpression was of no prognostic value (data not shown). By multivariate analysis, nodal status, Ep-CAM overexpression, tumour size and histologic grade, but not COX-2 overexpression, proved to be independent prognostic variables for overall survival. For disease-free survival, nodal status, tumour size and Ep-CAM overexpression, but not COX-2 overexpression, were independent prognostic factors (Table 2

**Table 2:** Multivariate analysis of various prognostic markers including Ep-CAM and COX-2 overexpression

	**DFS**			**OS**		
	***P***	**RR^a^**	**95% CI^b^**	***P***	**RR^a^**	**95% CI^b^**
Nodal status	0.001	2.3	1.4–3.8	<0.001	2.5	1.5–4.3
Ep-CAM overexpression	0.002	2.2	1.3–3.7	0.02	1.8	1.1–3.0
Tumour size						
<2 *vs* 2 >5 cm	NS^c^			NS^c^		
<2 vs >5 cm	0.004	3.7	1.5–8.9	0.003	3.7	1.5–8.9
Histological grade I+II *vs* III	NS^c^			0.01	2.0	1.2–3.4
Progesterone receptor	NS^c^			NS^c^		
Oestrogen receptor	NS^c^			NS^c^		
Her-2/neu overexpression	NS^c^			NS^c^		
COX-2 overexpression	NS^c^			NS^c^		

a Relative risk.

b Confidence interval.

c Not significant.

).

## DISCUSSION

Our study on 212 patients with invasive breast cancer confirms previous reports that COX-2 overexpression is rather frequent in this patient population (Soslow *et al*, 2000) and predicts a dismal prognosis for breast cancer patients (Ristimaki *et al*, 2002).

In experimental studies, COX-2 expression was related to local tumour invasiveness and metastatic potential (Tsujii *et al*, 1997). It has recently been demonstrated that COX-2 enhances angiogenesis, an effect that can be blocked by selective COX-2 inhibitors (Masferrer *et al*, 2000). Thus, COX-2 overexpression may provide a clinically useful biomarker for estimating tumour aggressiveness and patients' prognosis.

In our series, COX-2 overexpression was found to be absent in normal mammary gland epithelium surrounding malignant tissue. This observation is in keeping with recent data showing frequently higher COX-2 expression in various epithelial neoplasia compared with adjacent normal tissue (Soslow *et al*, 2000; Ristimaki *et al*, 2002).

Ep-CAM expression was found to correlate with cell proliferation and dedifferentiation in epithelial cells (de Boer *et al*, 1999). To date, little is known about the molecular mechanisms responsible for the regulation of the Ep-CAM gene. The highly significant association of COX-2 and Ep-CAM overexpression suggests a linkage between COX-2 and Ep-CAM signalling. Indeed, Tsujii and DuBois (1995) demonstrated that COX-2 can disrupt cell adhesion mediated by cadherins. Downregulation of cadherins in turn can upregulate Ep-CAM expression. Moreover, cytokines such as IFN*α* have been shown to upregulate both COX-2 and Ep-CAM expression in epithelial tumour cells (Bostrom *et al*, 2001; Flieger *et al*, 2001). Notably, no correlation was found between Her-2/neu and COX-2 overexpression. This finding is somewhat unexpected, since at least in colorectal cells, COX-2 can be upregulated by Her-2/neu receptor signalling (Vadlamudi *et al*, 1999). Taken together, upon validation in prospective studies, the combination of COX-2 and Ep-CAM expression may significantly improve the estimation of breast cancer prognosis. Beside this, COX-2 and Ep-CAM expression have come into focus as novel targets for therapeutic interventions in colorectal cancer. It remains to be seen whether COX-2 inhibitors and Ep-CAM directed monoclonal antibodies turn out to be efficacious for the treatment of other epithelial cancers such as breast carcinoma.
